# Adolescent Executive Dysfunction in Daily Life: Relationships to Risks, Brain Structure and Substance Use

**DOI:** 10.3389/fnbeh.2017.00223

**Published:** 2017-11-13

**Authors:** Duncan B. Clark, Tammy Chung, Christopher S. Martin, Brant P. Hasler, Douglas H. Fitzgerald, Beatriz Luna, Sandra A. Brown, Susan F. Tapert, Ty Brumback, Kevin Cummins, Adolf Pfefferbaum, Edith V. Sullivan, Kilian M. Pohl, Ian M. Colrain, Fiona C. Baker, Michael D. De Bellis, Kate B. Nooner, Bonnie J. Nagel

**Affiliations:** ^1^Department of Psychiatry, University of Pittsburgh, Pittsburgh, PA, United States; ^2^Department of Psychology and Psychiatry, University of California, San Diego, La Jolla, CA, United States; ^3^Center for Health Sciences, SRI International, Menlo Park, CA, United States; ^4^Department of Psychiatry and Behavioral Sciences, School of Medicine, Stanford University, Palo Alto, CA, United States; ^5^Department of Psychiatry, Duke University, Durham, NC, United States; ^6^Department of Psychology, University of North Carolina Wilmington, Wilmington, NC, United States; ^7^Department of Psychiatry, Oregon Health and Science University, Portland, OR, United States

**Keywords:** adolescents, executive functioning, neurocognitive testing, neuroimaging, alcohol

## Abstract

During adolescence, problems reflecting cognitive, behavioral and affective dysregulation, such as inattention and emotional dyscontrol, have been observed to be associated with substance use disorder (SUD) risks and outcomes. Prior studies have typically been with small samples, and have typically not included comprehensive measurement of executive dysfunction domains. The relationships of executive dysfunction in daily life with performance based testing of cognitive skills and structural brain characteristics, thought to be the basis for executive functioning, have not been definitively determined. The aims of this study were to determine the relationships between executive dysfunction in daily life, measured by the Behavior Rating Inventory of Executive Function (BRIEF), cognitive skills and structural brain characteristics, and SUD risks, including a global SUD risk indicator, sleep quality, and risky alcohol and cannabis use. In addition to bivariate relationships, multivariate models were tested. The subjects (*n* = 817; ages 12 through 21) were participants in the National Consortium on Alcohol and Neurodevelopment in Adolescence (NCANDA) study. The results indicated that executive dysfunction was significantly related to SUD risks, poor sleep quality, risky alcohol use and cannabis use, and was not significantly related to cognitive skills or structural brain characteristics. In multivariate models, the relationship between poor sleep quality and risky substance use was mediated by executive dysfunction. While these cross-sectional relationships need to be further examined in longitudinal analyses, the results suggest that poor sleep quality and executive dysfunction may be viable preventive intervention targets to reduce adolescent substance use.

## Introduction

Executive functioning is a broad construct comprised of behavioral competencies and cognitive skills, including attention, inhibition, mental flexibility, working memory, self-monitoring, planning, and emotional control (Chan et al., 2008). These self-control capabilities and higher order cognitive skills support the optimization of responding to environmental and personal challenges so as to maximize reward opportunities and achieve long-term goals (Best and Miller, 2010; Diamond and Lee, 2011). Problems in executive functioning in daily life (i.e., executive dysfunction) and cognitive skills assessed by performance-based testing may, in fact, reflect different constructs.

Characteristics reflecting executive dysfunction has been found to be associated with substance use disorder (SUD) risks and outcomes (Clark and Winters, 2002; Tarter et al., 2003; Clark et al., 2008). Pertinent indicators include dysregulation in cognitive (e.g., attention deficits), behavioral (e.g., impulsivity), and affective (e.g., affective lability) domains (Clark and Winters, 2002; Vanyukov et al., 2009; Clark et al., 2012). Most studies on these relationships have not explicitly and comprehensively measured executive dysfunction dimensions, however, leaving remaining questions on the extent to which SUD risks and outcomes may relate to specific dimensions or global executive dysfunction indicators.

Several studies have examined executive dysfunction in relationship to SUD risks and outcomes using a comprehensive multidimensional executive dysfunction measure, the Behavior Rating Inventory of Executive Function (BRIEF-SR: Gioia et al., 2002a,b; Roth et al., 2015). Expressly developed to measure eight executive dysfunction dimensions, the BRIEF has been demonstrated to be a valid and practical tool in school and clinical settings as well as in research (Egeland and Fallmyr, 2010; Roth et al., 2015). The BRIEF has been utilized in adolescent research to study executive dysfunction in relationship to SUD risks and outcomes, including disruptive behavior disorders (Wang et al., 2012; Long et al., 2015), sleep problems (Anderson et al., 2009; Caruso et al., 2014), academic achievement (Langberg et al., 2013; Samuels et al., 2016), performance-based cognitive tests (Shimoni et al., 2012; Long et al., 2015), brain structure and function (Clark et al., 2012; Ziegler et al., 2013; Zhai et al., 2015), and substance use (Clark et al., 2012). While comprehensively measuring executive dysfunction dimensions, most of these prior studies have had small sample sizes. Compared with prior studies, the present study includes a broader array of assessment domains with a considerably larger sample.

Among adolescents, poor sleep has been found to be associated with difficulties that may reflect executive dysfunction, such as inattention and depression (Millman, 2005). Limited research has specifically addressed the relationship between sleep quality and expressly measured executive dysfunction among adolescents. In 236 healthy adolescents (age 13–16 years: Anderson et al., 2009), increased sleepiness was significantly associated with more problems on dimensions including Working Memory, Task Planning, Orderliness, and Task Completion (i.e., BRIEF Metacognition) and not associated with Inhibitory Control, Flexibility, Emotional Control and Monitoring (i.e., BRIEF Behavioral Regulation). Sleep problems and circadian misalignment have been found to predict adolescent alcohol and other substance use (Hasler and Clark, 2013; Hasler et al., 2014, 2016, 2017). A study with a larger sample is needed to determine whether the relationships observed between executive dysfunction and poor sleep (Anderson et al., 2009) can be confirmed. Since poor sleep may influence executive dysfunction and executive dysfunction may, in turn, influence substance use, an examination of the relationships among these characteristics may suggest mechanisms, preventive intervention targets, and directions for future research.

The construct of executive functioning refers to behaviors in daily life as well as specific cognitive skills. A hypothesis about executive functioning in daily life is that variations in specific cognitive skills reflect the capabilities needed for effective functioning (Willcutt et al., 2005). To the extent that variations in objectively assessed cognitive skills correlate with executive dysfunction, such testing may be useful in depicting a mechanism for functional deficits. Relevant constructs assessed by performance-based cognitive testing include attention (e.g., Continuous Performance Test: Kurtz et al., 2001) and working memory (e.g., N-Back Test: Ragland et al., 2002). Several studies with small adolescent samples have noted, however, that performance-based cognitive tests and executive dysfunction ratings have little or no correspondence (Mahone et al., 2009; Cyders and Coskunpinar, 2012; Boschloo et al., 2014; Long et al., 2015; see Toplak et al., 2013 for review), with some exceptions (e.g., BRIEF and Working Memory by cognitive testing: Faridi et al., 2015). These studies suggest that cognitive testing of these constructs may not be particularly informative in understanding executive dysfunction in daily life. The present study examined these relationships in a large sample.

Executive dysfunction may reflect delays or deficits in neuromaturation. To the extent that variations in structural brain characteristics are found to correlate with executive dysfunction, such findings would support a hypothesis that specific observable brain characteristics reflect the neurobiological foundation for executive functioning. Prefrontal cortex (PFC) development has been hypothesized to be the neurobiological foundation for maturing executive functioning during adolescence, with the interaction of PFC with other functionally specialized brain regions critical for integrative executive functions (Spear, 2000). During adolescence, the maturation of executive cognitive skills occurs in parallel with increasing organization or integrity of white matter tracts projecting to PFC (Klingberg et al., 1999; Chambers et al., 2003; Takahashi et al., 2004; Lenroot and Giedd, 2006; Ashtari et al., 2007; Paus, 2010). The frontoparietal network (Fassbender et al., 2006), including tracts such as the superior longitudinal fasciculus (SLF) that connect the frontal and parietal cortex. The delay or disruption in the maturation of white matter integrity has been implicated as a neurobiological substrate for executive dysfunction (Lipton et al., 2009; Skranes et al., 2009; Clark et al., 2012). A few studies with adolescents have examined executive dysfunction, measured by BRIEF, in relationship to structural brain characteristics. Among 35 adolescents with SUD and 20 controls, worse executive dysfunction was significantly correlated with less PFC and parietal white matter integrity by diffusion tensor imaging fractional anisotropy (FA: Clark et al., 2012). Among 35 children and adolescents (Mahone et al., 2009), problems indicated on the BRIEF Working Memory scale were significantly correlated with smaller frontal gray matter volume, but not with temporal, parietal or occipital gray matter volume. In the NIH MRI study (Faridi et al., 2015), BRIEF Inhibitory Control was not significantly correlated with cortical gray thickness, while BRIEF Working Memory and Emotional Control showed greater cortical thickness associated with fewer problems only in the parahippocampal gyri. These studies do not clearly establish consistent relationships between adolescent executive dysfunction and structural brain characteristics, and the present larger study will clarifiy these relationships.

To the extent that executive dysfunction is found to correlate with the early development of risky substance use patterns, such findings would support the hypothesis that deficits in self-control reflected in executive dysfunction may contribute to SUD. In a few adolescent samples, executive dysfunction, indicated by BRIEF, have been found to be associated with substance involvement. BRIEF Global Composite (GEC) has been found to be significantly different among adolescents with SUD (*n* = 35) and control adolescents (*n* = 20), with 29% of the SUD group showing scores considered to indicate clinical problems (i.e., ≥70; Clark et al., 2012). In this sample, BRIEF GEC mediated the relationship between disruption of frontoparietal white matter integrity and cannabis symptoms. More everyday executive functioning problems indicated by BRIEF scores have also been observed in young adults with substance use (Hadjiefthyvoulou et al., 2012). More extensive study of these relationships is needed, particularly the examination of the relationship between executive dysfunction and the adolescent onset of risky substance use patterns.

This study examined these relationships at the initial assessment among subjects in National Consortium on Alcohol and Neurodevelopment in Adolescents (NCANDA), providing the opportunity to examine executive dysfunction in a relatively large, representative sample with a broad array of hypothetically related psychosocial, neurocognitive and brain measures. The aims of this study were to determine the relationships between executive dysfunction in daily life, measured by the Behavior Rating Inventory of Executive Function (BRIEF), cognitive skills and structural brain characteristics, and SUD risks, including a global SUD risk indicator, sleep quality, and risky alcohol and cannabis use. In addition to bivariate relationships, multivariate models were tested.

## Methods

### Participants

NCANDA participants were 831 youth ranging in age from 12 to 21 years. NCANDA examines adolescent neurodevelopmental risks for and outcomes of alcohol use in a large, multisite, accelerated longitudinal design (see Brown et al., 2015 for additional details). NCANDA subjects were recruited from five sites: Duke University, University of Pittsburgh, Oregon Health and Science University, University of California, San Diego, and SRI International. For this analysis, 10 subjects were missing BRIEF, and 4 were excluded for invalid results (see below). The demographic characteristics were as follows: age: mean: 16.2 years, s.d. 2.5, range: 12.0–21.9; female: *n* = 417, male: *n* = 400; race: white: *n* = 586 (71.7%), African American: 95 (11.6%), Asian: *n* = 62 (7.6%); Native American: *n* = 3 (<1%); Pacific Islander: *n* = 4 (<1%); multiple: *n* = 67 (8.2%); ethnicity: Hispanic: *n* = 96 (11.8%), non-Hispanic: *n* = 721 (88.2%). These race and ethnicity proportions are similar to the U.S. population (Brown et al., 2015). Most subjects (*n* = 692, 83%) had little or no alcohol use history, and a subsample (*n* = 139, 17%) had a history of risky alcohol use based on exceeding established alcohol use thresholds (National Institute on Alcohol Abuse and Alcoholism, 2011). Youth at increased risk for problematic alcohol use, based on one or more risk factors including early alcohol use, family SUD history, disruptive behavior disorder symptoms, or two or more anxiety or depression symptoms, comprised approximately 50% of the sample. The institutional review board at each site approved the study. Adult participants consented to participate, and minors provided assent with parental or legal guardian consent.

### Design

NCANDA uses an accelerated longitudinal design (Duncan et al., 1996, 2006), sampling subjects from a broad age span with subsequent follow-up assessments to characterize development across an expansive period. At the initial assessment, youth completed a comprehensive assessment of substance use, psychiatric symptoms and diagnoses, personality factors, and functioning in major life domains (Brown et al., 2015); a neurocognitive battery (Sullivan et al., 2016), and a neuroimaging assessment with structural and diffusion tensor imaging (Pfefferbaum et al., 2016; Pohl et al., 2016). One parent of each youth also completed an assessment. These analyses utilized the initial assessment data.

### Measures

#### BRIEF

Behavior Rating Inventory of Executive Function-Self-Report Version (BRIEF-SR: Baron, 2000; Gioia et al., 2002a,b; Guy et al., 2004; Roth et al., 2015): The BRIEF-SR is an 80-item adolescent self-report behavior rating scale of purposeful, goal-directed, problem-solving behavior. For each item, the subject is asked: “Over the past 6 months, how often has each of the following behaviors been a problem?” The response choices are “Often” (3 points), “Sometimes” (2 points) and “Never” (1 point). The instrument yields a summary score, the Global Executive Composite (GEC), two composite indices, and eight scales: (1) The ***Inhibitory Control*** scale (13 items) assesses inhibitory control and impulsivity (e.g., “*I have problems waiting my turn,” “I don't think of consequences before acting,” “I get out of control more than my friends”*). (2) The ***Flexibility*** scale (10 items), including behavioral and cognitive characteristics, assesses the ability to make transitions and flexibly solve problems (e.g., “*I have trouble changing from one activity to another”*). (3) The ***Emotional Control*** scale (10 items) assesses the ability to modulate emotional responses in response to situational demands (e.g., “*I overreact to small problems,” “I get upset easily”*). (4) The ***Monitoring*** scale (5 items) assesses self-awareness of interpersonal strengths and weaknesses (e.g., “*I don't know when my actions bother others”*). (5) The ***Working*** ***Memory*** scale (12 items) assesses holding information in mind for the purpose of completing a task (e.g., “*I forget instructions easily,” “I have trouble with jobs or tasks that have more than one step”*). (6) The Task ***Planning*** scale (Plan: 13 items) assesses planning steps to complete a task and the anticipation of future consequences (e.g., “*I don't plan ahead for school assignments,” “I don't think ahead about possible problems” “I have trouble carrying out the things that are needed to reach a goal, such as saving money for special needs, studying to get good grades, etc.”*). (7) The ***Organization*** scale (Organize: 7 items) assesses the ability to keep work and school materials organized (e.g., “*My backpack/schoolbag is disorganized”*). (8) The ***Task Completion*** assesses the ability to complete school or work in a timely fashion by 10 items (e.g., “*I have difficulty finishing a task on my own”*). The ***Behavioral Regulation Index (BRI)*** combines Inhibit, Shift, Emotional Control, and Monitor scales (internal consistency: 0.96). The ***Metacognition Index*** ***(MCI)*** is comprised of Initiate, Working Memory, Plan and Organize, Task Monitor, and Organization of Materials subscales (internal consistency: 0.72). In addition, validity scales include the ***Inconsistency*** scale, which determines whether the subject responded in a consistent manner by comparing 10 pairs of similar items, and the ***Negativity*** scale (10 items), which determines whether the subject responds to selected items in an unusually negative manner. Raw scores are converted to age indexed t-scores, with higher scores indicating more problems. Subjects with missing (*n* = 10) or invalid (*n* = 4) BRIEF scores were excluded from all analyses.

#### Risk factors for alcohol use disorder (AUD)

The NCANDA sample (Brown et al., 2015) was configured to include approximately 50% of the sample at higher risk for problematic alcohol use based on screening evidence for one of the following: (1) early alcohol use (i.e., first standard drink before age 15 years old); (2) a family history of alcohol or other substance problems; (3) endorsement of one or more conduct disorder or antisocial personality disorder symptoms or *t* score ≥ 60 on the externalizing score of the Achenbach system of Empirically Based Assessments (ASEBA: Achenbach and Rescorla, 2001a,b); (4) endorsement of two or more internalizing symptoms or *t* ≥ 60 on ASEBA internalizing. ***Risk Density*** was calculated as the sum of the presence (1) or absence (0) of each of these characteristics (range: 0–4).

#### Subjective sleep quality

The Pittsburgh Sleep Quality Index (PSQI: Buysse et al., 1989) has been shown to be comprised of two factors, sleep efficiency and perceived sleep quality (Mollayeva et al., 2016). The PSQI has been validated in adolescent samples (de la Vega et al., 2015) and has been commonly used in adolescent research (e.g., Noone et al., 2014). The perceived sleep quality factor includes a subjective sleep quality item which correlates highly with the factor score and loads more strongly on this factor than do other items (Mollayeva et al., 2016). The subjective sleep quality item asks “During the past month, how would you rate your sleep quality overall?” with response options “very good,” “fairly good,” “fairly bad,” and “very bad.”

#### Performance based cognitive tests

The NCANDA performance based cognitive testing protocol was designed to assess functional domains relevant for alcohol involvement risks and outcomes (see Sullivan et al., 2016 for details). The NCANDA cognitive and motor battery assessed eight domains: general ability, attention, abstraction, emotion, working memory, balance, and motor speed (Sullivan et al., 2016, 2017). Most of these domains were assessed with computer-administered WebCNP tests (Gur et al., 2010). For examining cognitive skills that may be pertinent to executive dysfunction here, the domains of attention, emotion and working memory are particularly pertinent, and the examination of general ability may provide information on the global effects of problems in this arena. The functional domains and tests included here were: (1) Attention: Continuous Performance Test (Kurtz et al., 2001); (2) Emotion: Emotion Recognition Test (Gur et al., 2002), Measured Emotion Differentiation (Fossati, 2012); (3) Working Memory: Short Fractal N-Back Test (Ragland et al., 2002); and (4) General Ability: Vocabulary Test (Lee et al., 2014), WRAT-4 Math Calculations and Word Reading (Wilkinson and Robertson, 2006). These computer administered WebCNP tests were used to generate domain specific accuracy and speed (response time) *z* scores. In prior analyses, older age was associated with better scores on attention, emotion, and general ability (Sullivan et al., 2016), so age was used as a covariate in these analyses.

### MRI acquisition and analysis

The NCANDA neuroimaging battery includes structural indices (Pfefferbaum et al., 2016) and diffusion tensor imaging (DTI: Pohl et al., 2016), and more detailed descriptions of the protocols may be found in those publications. Briefly, T1-weighted, 3D images were collected in the sagittal plane on systems from two manufacturers: 3T General Electric (GE) Discovery MR750 at three sites and 3T Siemens TIM TRIO scanners at two sites. The GE sites used an Array Spatial Sensitivity Encoding Technique (ASSET) for parallel and accelerated imaging with an 8-channel head coil and acquired an Inversion Recovery-Spoiled Gradient Recalled (IR-SPGR) echo sequence. The Siemens sites used a 12-channel head coil and parallel imaging and temporal acceleration with iPAT and acquired an MPRAGE sequence. Each site scanned the ADNI phantom on each day that participants were scanned. Analysis proceeded via the NCANDA NeuroInformatics Platform (Rohlfing et al., 2014) and involved skull stripping applied to the extracted maps. The SRI24 atlas-based analysis pipeline was used to identify intracranial volume (ICV), supratentorial volume (svol), and pons, corpus callosum, subcortical white matter (including the centrum semiovale), and lateral ventricular volumes. FreeSurfer (Dale et al., 1999) was used on skull-stripped data to create bilateral surface area, volume, and thickness of frontal, temporal, parietal, occipital, cingulate cortices derived from the Desikan-Killiany regions-of-interest (ROI) scheme (Desikan et al., 2006) plus the insular cortex. Volume was expressed in cc, surface area in cm^2^, and thickness in mm. All ROIs were adjusted for linear scaling factors from the ADNI phantom (Clarkson et al., 2009). To acquire Diffusion-Weighted Images (DWI, a.k.a. diffusion tensor imaging or DTI), GE and Siemens sites applied a 2D Axial Spin Echo, Echo-Planar protocol, as well as a reverse phase acquisition of the 2D Axial Spin Echo-Planar protocol for B0-field inhomogeneity spatial distortion correction. To achieve common anatomical coordinates across subjects, each subject's fractional anisotropy (FA) data set was registered to the FA channel of the SRI24 atlas. Cortical volume and thickness were smaller with increasing age, and cortical volumes greater in males than females (Pfefferbaum et al., 2016). For DTI indices, increasing age was associated with higher FA and lower diffusivity measures (Pohl et al., 2016). Consequently, statistical analyses on structural brain characteristics in the present study were controlled for age and sex.

### Substance use

Past and recent alcohol and other substance use were determined by the Customary Drinking and Drug Use Record (CDDR: Brown et al., 1998). The measure includes items on alcohol and marijuana use, including use frequency in the past year, and the maximum number of drinks in a drinking episode during the past year. At **Risk Alcohol Use:** Alcohol use frequency in the past year has been found to useful for identifying use patterns associated with alcohol use disorder (AUD) among adolescents (e.g., Chung et al., 2012; Clark et al., 2016). The National Institute on Alcohol Abuse and Alcoholism (NIAAA) Alcohol Screening and Brief Intervention for Youth: A Practitioner's Guide (NIAAA Youth Guide) recommends age specific stratified past year alcohol use frequency thresholds to define low, moderate and high risk for AUD. Moderate Risk is defined at age 12–15 as: ≥1 days; ages 16–17: ≥3 days; ages 18: ≥12 days. High Risk is defined at age 12–15 as: ≥3 days; age 16: ≥12 days; age 17: ≥24 days; age 18: ≥52 days. Subjects below these thresholds were classified as Low Risk. **Binge Alcohol Use:** While the traditional definition of a drinking binge has typically been applied across development, binge definitions appropriate for younger adolescents have been developed to account for smaller body size among youth (Donovan, 2009). Using the “lifetime greatest number of drinks” response, age-specific binge thresholds were calculated as follows: ages 9 to 13 years: ≥3 drinks; 14 or 15 years: ≥4 for males, ≥3 for females; 16 or 17 years: ≥5 for males, ≥3 for females.

### Statistical analyses

Overall NCANDA data management has been described (Rohlfing et al., 2014). (NCANDA Data releases used in these analyses: 000001_V1; 00010_V3; 00011; 00012_V2.) The analyses presented to test hypotheses were by analysis of variance (ANOVA) for categorical variables or Pearson correlations for continuous variables. Covariates for all analyses were age, sex and SES. A sequential procedure (Ludbrook, 1998) was used to reduce Type 1 error or family wise error rate, first testing the global hypothesis represented by the BRIEF Global Composite (i.e., GEC), followed by tests examining the BRIEF indices and scales with Šidák corrections (Šidák, 1967; Ludbrook, 1998). For analyses examining BRIEF composite, indices and scales, BRIEF GEC was examined first (*p* < 0.05) and, where the relationship between GEC and the dependent measure were significant, relationships with Behavioral Regulation Index (BRI) and Metacognition Index (MCI), then the eight scales, were interpreted. Corrections for multiple tests were implemented as follows: Behavioral Regulation Index or BRI and Metacognition Index or MCI: α = 0.05 for 2 tests: *p* < 0.025; 8 scales: α = 0.05 for 8 tests: *p* < 0.0064). To reduce type 1 error in sets of analyses examining correlations between multiple continuous variables (i.e., cognitive test scores and brain structure variables) and BRIEF GEC, the Šidák procedure was applied, in which the overall number of comparisons was taken into consideration to determine the significance threshold (Cognitive skill domains, with scores for accuracy and speed: α = 0.05/2 or *p* < 0.025; MR Structural Gray Indices: α = 0.05/18 or *p* < 0.0028; DTI: α = 0.05/32 or *p* < 0.0016). For variables with significant relationships with BRIEF GEC, multivariate models were constructed to test mediation hypotheses (Baron and Kenny, 1986).

## Results

### BRIEF description

For subjects with valid BRIEF data (*n* = 817), mean scale t-scores ranged from 43.5 ± 8.6 (BRIEF Inhibitory Control) to 46.1 ± 9.9 (BRIEF Task Completion), with composite t-scores also in this range (BRI: 43.6 ± 9.4; MCI: 44.6 ± 10.6; GEC: 43.9 ± 10.4).

### Demographic characteristics

BRIEF composite scores did not significantly differ by gender (e.g., GEC: F: 44.0 ± 11.1; M: 43.8 ± 9.6; *t* = 0.2, d.f. 815, *p* = 0.8). BRIEF GEC was not significantly correlated with age (*r* = 0.06, *n* = 817, *p* = 0.10). BRIEF GEC was significantly correlated with SES (*r* = −0.15, *n* = 731, *p* < 0.001). Race (White: *n* = 586, 43.7 ± 10.1; African American: *n* = 95, 43.3 ± 11.7; Asian: *n* = 62, 43.9 ± 8.9; Other: *n* = 67, 46.6 ± 12.0) was not significantly associated with BRIEF GEC (*F* = 1.7, d.f. 3,806, *p* = 0.16). Ethnicity (Non-Hispanic: *n* = 721, 43.2 ± 11.9; Hispanic: 49.1 ± 11.9) was significantly associated with BRIEF GEC (*F* = 28.4, 1,815, *p* < 0.001). To a substantial extent, this relationship was accounted for by SES (multivariate model: *n* = 725; SES: *F* = 13.1, *p* < 0.001; Race: *F* = 1.6, *p* = 0.6; Ethnicity: *F* = 4.9, *p* = 0.03).

### NCANDA risk density

**Figure 1:**
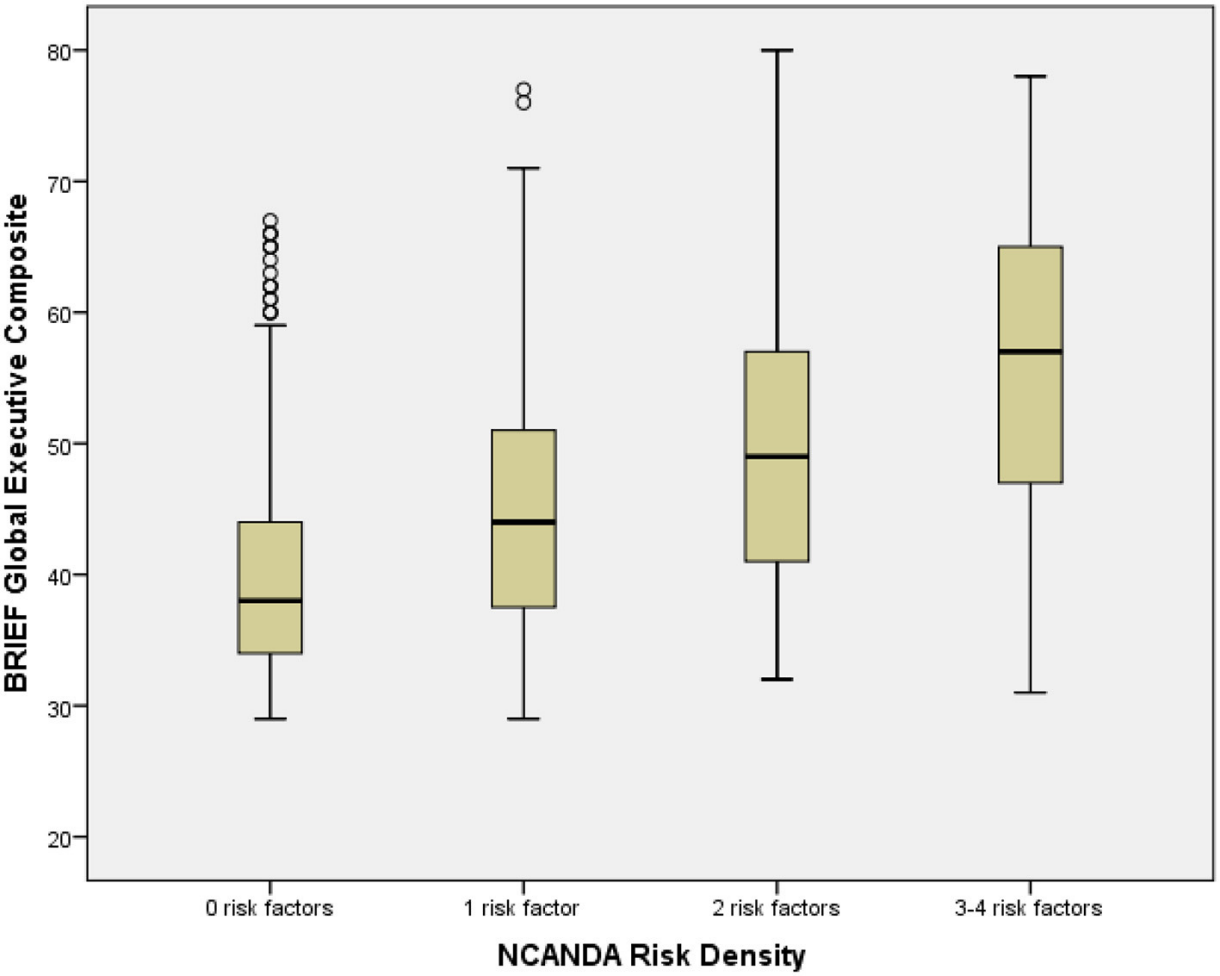
Box plot of BRIEF global composite by NCANDA risk density categories. The figure shows box plots for NCANDA Risk Density categories. The box plot represents the interquartile range (IQR), with the shaded box representing the second and third quartiles and center demarcations indicating the median. The whiskers represent the maximum and minimum values up to 1.5 × IQR, and the circles represent values beyond 1.5 × IQR.

Risk Density (Table 1) was determined by summing the number of risks present (range: 0–4; 0: *n* = 381, 46.6%; 1: 276, 33.8%; 2: 119, 14.6%; 3: 37, 4.5%; 4: 4, 0.5%). Due to few participants with 4 risk characteristics, participants with 3 or 4 risk factors were combined for analyses. Risk Density was significantly associated with BRIEF GEC, with higher Risk Density associated with greater dysfunction (Table 1; Figure 1). All BRIEF indices and scales were similarly significantly related to Risk Density. Abnormal BRIEF GEC scores (≥70), present in 2% of the sample, were associated with Risks (0 Risks, 0%; 1 risk, 2%, 2 risks, 4%, 3 or 4 Risks, 20%: Likelihood Ratio χ^2^ = 68.0, d.f. = 3, *p* < 0.001). Risk Density was significantly associated with At Risk Alcohol Use (*F* = 40.5, *p* < 0.001), Binge Alcohol Episodes (*F* = 7.4, *p* < 0.01), and Marijuana Use (*F* = 10.3, *p* < 0.01) (Table 8).

**Table 1:** BRIEF global composite, indexes and scales by NCANDA risk density categories.

	**0**	**1**	**2**	**3–4**	***F***
	***n* = 381**	***n* = 276**	***n* = 119**	***n* = 41**	
Inhibitory control	40.8 ± 7.2	44.5 ± 8.2	46.9 ± 9.0	54.0 ±12.0	37.3^c^
Flexibility	42.7 ± 9.3	46.9 ± 10.1	51.1 ± 12.4	55.4 ± 11.1	28.9^c^
Emotional control	42.4 ± 7.3	46.2 ± 8.5	50.9 ± 10.6	53.3 ± 11.5	36.6^c^
Monitoring	42.5 ± 7.6	45.3 ± 8.6	50.2 ± 9.2	54.1 ± 10.1	33.5^c^
Behavioral regulation	40.1 ± 7.7	44.7 ± 8.7	49.5 ± 10.2	55.0 ± 11.0	51.3^b^
Working memory	41.9 ± 8.4	46.9 ± 10.0	50.1 ± 10.8	56.9 ± 12.0	40.0^c^
Task plan	41.9 ± 8.7	46.7 ± 9.9	50.6 ± 1.5	56.7 ± 10.8	39.9^c^
Orderly	43.0 ± 8.7	46.9 ± 9.5	49.2 ± 10.0	54.4 ± 11.2	24.4^c^
Completion	42.3 ± 7.3	47.5 ± 9.8	51.4 ± 11.0	57.9 ± 12.7	47.6^c^
Metacognition	40.9 ± 8.5	46.6 ±10.2	50.4 ± 11.2	57.7 ± 11.9	50.1^b^
Global composite	39.9 ± 8.2	45.0 ± 9.6	50.0 ± 10.9	56.5 ± 11.7	55.8^a^

*d.f. 3, 724; covariates: sex, age, SES*.

a *p < 0.05 for test of GEC*.

b *p < 0.025 for 2 tests of Indexes*.

c *p < 0.0064 for 8 tests of Scales*.

### Sleep quality

**Figure 2:**
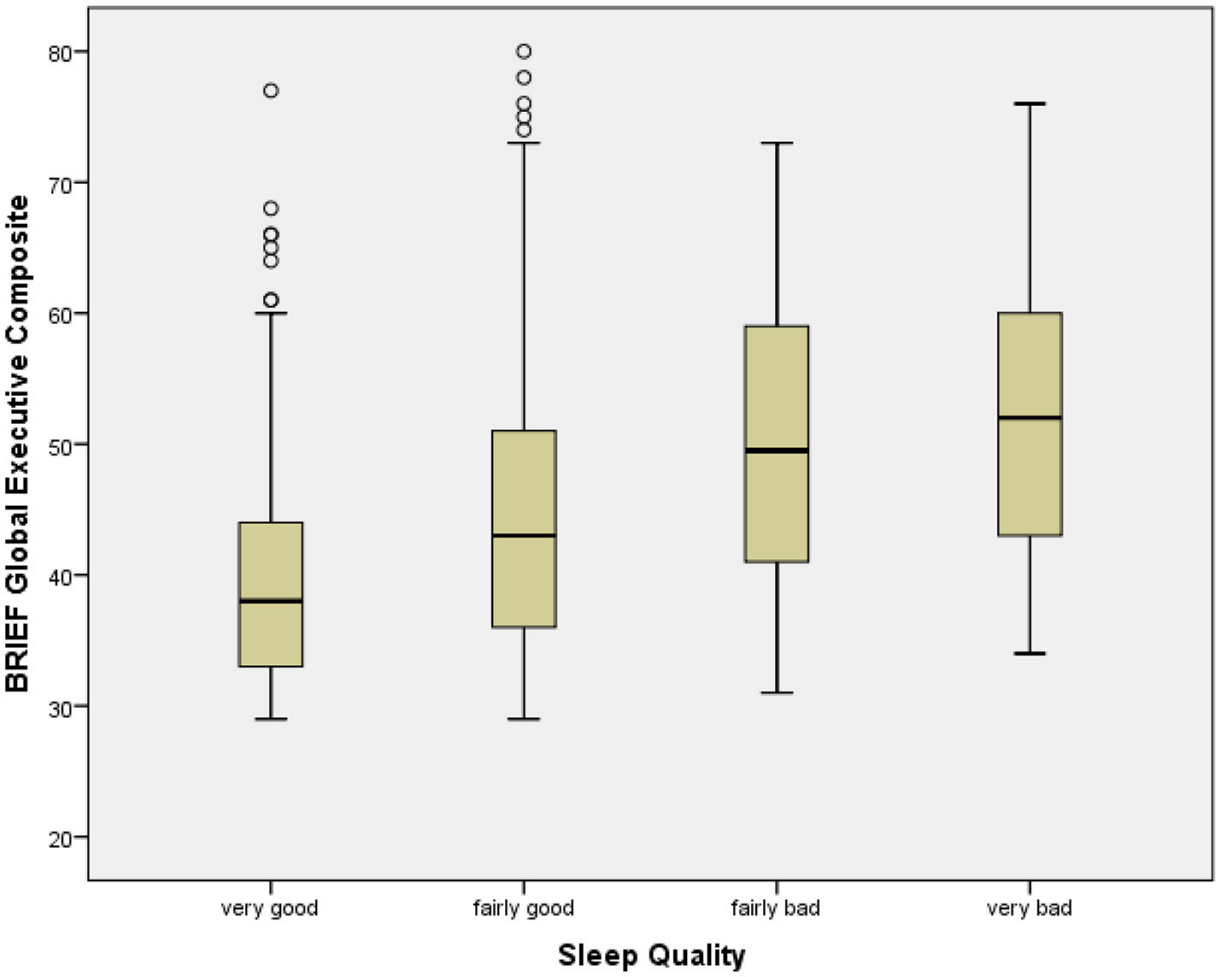
Box plot of BRIEF global executive composite by sleep quality categories. The figure shows box plots for Sleep Quality categories. The box plot represents the interquartile range (IQR), with the shaded box representing the second and third quartiles and center demarcations indicating the median. The whiskers represent the maximum and minimum values up to 1.5 × IQR, and the circles represent values beyond 1.5 × IQR.

Sleep Quality was significantly associated with BRIEF GEC, with poorer slep quality associated with greater dysfunction (Table 2; Figure 2). All BRIEF indices and scales were similarly significantly related to Sleep Quality. Sleep Quality was significantly associated with At Risk Alcohol Use (*F* = 5.6, *p* < 0.05), Binge Alcohol Episodes (*F* = 3.9, *p* < 0.05), and Marijuana Use (*F* = 5.8, *p* < 0.05) (Table 8).

**Table 2:** BRIEF global composite, indexes and scales by sleep quality categories.

	**Very good**	**Fairly good**	**Fairly bad**	**Very bad**	***F***
	***n*** = **218**	***n*** = **430**	***n*** = **71**	***n*** = **12**	
Inhibitory control	41.0 ± 7.1	44.0 ± 8.7	48.3 ± 10.7	47.2 ± 9.8	14.5^c^
Flexibility	42.7 ± 9.6	46.5 ± 10.7	51.5 ± 10.6	53.8 ± 15.4	15.4^c^
Emotional control	42.9 ± 8.1	46.2 ± 9.3	48.2 ± 9.5	49.5 ± 9.8	9.6^c^
Monitoring	42.3 ± 7.7	45.9 ± 8.9	48.6 ± 9.3	48.5 13.2	12.8^c^
Behavioral regulation	40.4 ± 8.2	44.4 ± 9.4	48.9 ± 10.3	49.7 ± 11.8	18.7^b^
Working memory	42.0 ± 9.3	46.0 ± 9.8	52.3 ± 11.9	54.9 ± 11.9	24.2^c^
Task plan	41.3 ± 9.3	46.0 ± 9.8	53.3 ± 11.9	53.5 ± 11.3	28.5^c^
Orderly	42.2 ± 7.9	46.5 ± 9.7	50.6 ± 11.3	55.4 ± 9.6	20.4^c^
Task completion	42.9 ± 8.3	46.2 ± 9.6	54.4 ± 11.8	54.3 ± 12.5	27.1^c^
Metacognition	40.9 ± 9.2	45.5 ± 10.0	53.3 ± 12.2	55.3 ± 12.6	31.8^b^
Global composite	39.9 ± 9.0	44.7 ± 10.3	51.4 ± 11.8	52.9 ± 12.4	28.3^a^

*d.f. 2, 723; covariates: sex, age, SES*.

a *p < 0.05 for test of BRIEF GEC*.

b *p < 0.025 for 2 tests of Indexes*.

c *p < 0.0064 for 8 tests of Scales*.

### Cognitive skill domain scores

Cognitive Skill Domain Scores were not significantly correlated with BRIEF GEC (Table 3). Note that, while a correction for two tests per domain (i.e., accuracy and speed) was thought applicable, a less stringent threshold of *p* < 0.05 did not change the interpretation of the results. Since there may be some interest in the observed correlations for specific variable pairs (e.g., Working Memory by BRIEF scale by Cognitive Skill Domain), the full table of analyses is presented.

**Table 3:** BRIEF global composite, indexes and scales by cognitive skill domain scores.

	**Attention**		**Emotion recognition**		**Working memory**		**General abiliity**	
	**a**	**s**	**a**	**s**	**a**	**s**	**a**	**s**
Inhibitory control	−0.08	−0.01	−0.01	0.13	0.01	0.00	−0.04	0.08
Flexibility	−0.02	−0.04	−0.02	0.03	0.02	−0.02	−0.01	0.02
Emotional control	−0.03	−0.01	0.03	0.05	0.04	0.02	−0.04	0.01
Monitoring	−0.03	−0.05	−0.03	0.07	−0.02	−0.02	−0.04	0.04
Behavioral regulation	−0.05	−0.03	−0.01	0.08	0.02	0.00	−0.04	0.04
Working memory	−0.05	−0.03	0.03	0.06	0.00	0.00	−0.04	0.04
Task planning	−0.04	−0.03	0.04	0.09	0.04	−0.02	0.01	0.07
Organization	0.01	−0.03	0.06	0.06	0.03	−0.02	0.01	0.07
Task completion	−0.05	−0.06	0.01	0.05	0.00	−0.05	−0.13	−0.02
Metacognition	−0.04	−0.03	0.04	0.07	0.02	−0.03	−0.04	0.05
Global composite	−0.05	−0.03	0.02	0.08	0.02	−0.01	−0.04	0.05

*A, accuracy; s, speed; Pearson correlations [r]; d.f. 707; covariates: sex, age, SES*.

*With corrections for multiple comparisons, none of the above statistical test reached statistical significance*.

### Cortical structure

Using a correction for multiple comparisons for tests across the BRIEF GEC by structural variables matrix (i.e., 18 tests), BRIEF GEC was not significantly correlated with gray matter volume, cortical thickness or cortical surface area in frontal temporal, parietal, occipital, cingulate, or insula regions (Table 4).

**Table 4:** BRIEF global composite by MR structural gray segment volume, thickness and surface area.

	**Volume**	**Thickness**	**Surface area**
Frontal	−0.01	−0.04	0.02
Temporal	0.02	0.00	0.04
Parietal	0.01	0.00	0.03
Occipital	−0.02	−0.01	0.00
Cingulate	0.00	−0.02	0.02
Insula	−0.03	−0.05	−0.01

*Pearson correlations [r]; d.f. 707; covariates: sex, age, SES*.

*With corrections for multiple comparisons, none of the above statistical test reached statistical significance*.

### DTI

Using a correction across the presented BRIEF GEC by structural variables (i.e., 32 tests), BRIEF GEC was not significantly correlated with DTI indices for fasciculi and tracts by region or DTI variable (Table 5).

**Table 5:** BRIEF global composite by DTI indices for fasciculi and tracts.

	**FA**	**MD**	**L1**	**LT**
**FASCICULI**				
Superior longitudinal	−0.07	0.08	0.03	0.04
Superior frontal-occipital	−0.07	0.00	−0.06	0.02
Sagittal stratum	0.01	0.01	0.04	−0.02
Uncinate	−0.09	0.08	0.00	0.08
**LIMBIC TRACTS**				
Fornix	0.01	0.01	0.02	0.00
Striatia terminalis	0.01	0.06	0.09	−0.01
Anterior middle cingulum	−0.03	0.11	0.09	0.03
Inferior cingulum	0.01	0.00	0.01	−0.01

*FA, fractional anisotrophy; MD, mean diffusivity; L1, axial diffusivity; LT, radial diffusivity. Pearson correlations [r]; d.f. 703; covariates: sex, age, SES. With corrections for multiple comparisons, none of the above statistical test reached statistical significance*.

### Alcohol and cannabis use

***At Risk Alcohol Use Category*** was determined by the past year alcohol use days frequency thresholds described in the National Institute on Alcohol Abuse and Alcoholism Youth Guide (2011). Overall (Table 6), 83% (*n* = 676) were Low Risk, 12% (*n* = 95) were Moderate Risk, and 5% (*n* = 41) were High Risk. At Risk Alcohol Use Category was significantly related to BRIEF GEC (*F* = 7.0, d.f. 2,723, *p* = 0.001), with higher risk associated with more problems. At Risk Alcohol Use Category was significantly associated with BRIEF BRI and MCI, and scales including Inhibitory Control, Emotional Control, and Task Planning.

**Table 6:** At Risk Alcohol Use Frequency x BRIEF scales.

	**Low risk**	**Moderate risk**	**High risk**	***F***
	***n* = 616**	***n* = 82**	***n* = 31**	
Inhibitory control	43.1 ± 8.4	46.3 ± 10.7	47.3 ± 9.0	8.1^c^
Flexibility	45.7 ± 10.8	48.0 ± 11.4	48.1 ± 11.7	2.3
Emotional control	45.1 ± 9.1	48.9 ± 9.2	47.1 ± 10.6	7.3^c^
Monitoring	44.8 ± 8.8	47.3 ± 10.0	47.6 ± 8.8	3.7
Behavioral regulation	43.3 ± 9.5	47.1 ± 10.5	46.7 ± 10.3	7.4^b^
Working memory	45.1 ± 10.3	48.6 ± 11.7	48.5 ± 9.4	5.4
Task plan	45.0 ± 10.4	47.9 ± 11.0	51.8 ± 11.0	8.0^c^
Orderly	45.5 ± 9.8	47.5 ± 10.1	48.9 ± 9.0	2.4
Task completion	45.7 ± 10.0	49.2 ± 11.0	48.1 ± 11.4	3.9
Metacognition	44.5 ± 10.7	48.0 ± 11.6	49.3 ± 10.5	5.9^b^
Global composite	43.5 ± 10.5	47.5 ± 11.2	48.1 ± 10.6	7.0^a^

*d.f. 2, 723; covariates: sex, age, SES*.

a *p < 0.05 for test of BRIEF GEC*.

b *p < 0.025 for 2 tests of Indices*.

c *p < 0.0064 for 8 tests of Scales*.

#### Age defined binge alcohol use

Past year age defined binge alcohol use (Table 7) was present in 129 subjects (15.9%). Including sex, age and SES as covariates, Age Defined Binge Alcohol Use was significantly related to BRIEF GEC (*F* = 11.1, d.f. 1,721, *p* = 0.001), with the presence of at least one binge in the past year associated with more problems. Age Defined Binge Alcohol Use was associated with more problems on BRIEF BRI and MCI, as well as on the including Inhibitory Control scale.

**Table 7:** Past year age defined binge and marijuana use × BRIEF scales.

	**Age defined binges**			**Marijuana use**		
	**No *n* = 626**	**Yes *n* = 100**	***F***	**No *n* = 621**	**Yes *n* = 104**	***F***
Inhibitory control	43.2 ± 8.6	46.1 ± 9.6	11.1^c^	43.1 ± 8.5	46.5 ± 9.9	12.2^c^
Flexibility	45.7 ± 10.7	48.0 ± 11.4	4.4	45.6 ± 10.6	48.3 ± 11.5	4.9
Emotional control	45.3 ± 9.5	46.9 ± 9.5	3.4	45.1 ± 9.1	48.1 ± 9.3	8.4^c^
Monitoring	44.8 ± 8.8	47.3 ± 9.8	6.5	44.8 ± 8.8	47.4 ± 9.3	5.5
Behavioral regulation	43.4 ± 9.4	46.3 ± 9.9	8.7^b^	43.2 ± 9.4	47.0 ± 10.2	11.0^b^
Working memory	45.3 ± 10.5	47.4 ± 9.5	4.4	45.1 ± 10.4	48.0 ± 10.1	5.3
Task plan	45.1 ± 10.4	48.1 ± 10.7	5.5	45.0 ± 10.5	48.6 ± 10.1	7.5^c^
Orderly	45.4 ± 9.7	48.1 ± 9.6	3.7	45.3 ± 9.8	48.2 ± 9.3	4.5
Task completion	45.8 ± 9.8	48.2 ± 11.1	3.7	45.8 ± 10.0	48.3 ± 10.4	2.7
Metacognition	44.7 ± 10.6	47.6 ± 10.8	5.6^b^	44.6 ± 10.7	48.8 ± 10.3	6.2^b^
Global composite	43.6 ± 10.4	46.8 ± 10.6	7.4^a^	43.5 ± 10.4	47.4 ± 10.5	9.1^a^

*Binge: d.f. 1, 721; covariates: sex, age, SES; Marijuana: d.f. 1, 720*.

a *p < 0.05 for test of Composite*.

b *p < 0.025 for 2 tests of Indexes*.

c *p < 0.0064 for 8 tests of Scales*.

#### Cannabis use

Past year cannabis use was present in 123 subjects (Table 7: 15.2%). Cannabis Use was significantly related to BRIEF GEC (*F* = 9.1, d.f. 1,720, *p* = 0.003), with the presence of cannabis use in the past year associated with more problems. Cannabis Use was associated with more problems on BRIEF BRI and MCI, as well as scales including Inhibitory Control, Emotional Control, and Task Planning.

#### Multivariate models

For variables shown to be significantly related to BRIEF GEC (i.e., NCANDA Risk Density and Sleep Quality), multivariate models were tested (Table 8). NCANDA Risk Density was significantly related to Past Year At Risk Alcohol Use Frequency, Age Defined Binge Alcohol Use and Cannabis Use (i.e., substance use). In multivariate models including BRIEF GEC, the relationship between Risk Density and substance use variables diminished but remained significant, suggesting that mediation was not demonstrated. Sleep Quality was significantly related to substance use variables. In multivariate models including BRIEF GEC, the relationship between Sleep Quality and substance use variables was not statistically significant, suggesting mediation. In a model with NCANDA Risk Density, Sleep Quality and BRIEF GEC, the relationship between NCANDA Risk Density and substance use remained significance while Sleep Quality was not significantly related to substance use.

**Table 8:** Contributions to ANOVA models of substance use outcomes.

	**At Risk Alcohol**		**Binge alcohol**		**Cannabis use**	
**Tested covariates**	***F***	**(*R*^2^)**	***F***	**(*R*^2^)**	***F***	**(*R*^2^)**
BRIEF alone	13.7^***^	(0.070)	7.4^**^	(0.126)	10.3^**^	(0.141)
Risk alone	40.5 ^***^	(0.102)	14.1^***^	(0.134)	28.6^***^	(0.162)
Risk with BRIEF	27.6^***^	(0.103)	8.2^**^	(0.134)	19.1^***^	(0.162)
BRIEF with risk	1.3		1.5		1.1	
Sleep alone	5.6^*^	(0.059)	3.9^*^	(0.122)	5.8^*^	(0.136)
Sleep with BRIEF	1.6	(0.070)	1.4	(0.126)	2.1	(0.143)
BRIEF with sleep	9.7^**^		4.8^*^		6.6^**^	
BRIEF with risk and sleep	0.7	(0.103)	0.8	(0.135)	0.4	(0.163)
Risk with BRIEF and sleep	27.0^***^		7.8^**^		18.5^***^	
Sleep with BRIEF and risk	1.0		1.1		1.6	

Adjusted R^2^ for the model; F-statistic with d.f. 1, 701; covariates: sex, age, SES;

* *p < 0.05*,

** *p < 0.01*,

*** *p < 0.001; BRIEF, BRIEF Global Composite; Risk, NCANDA Risk Density; Sleep, Sleep Quality; At Risk Alcohol, At Risk Alcohol Frequency (Past Year); Binge Alcohol, Age Defined Binge Alcohol Use (Past Year); Marijuana Use, Marijuana Use (Past Year)*.

## Discussion

In NCANDA, BRIEF (Guy et al., 2004) was utilized as an ecologically valid complement to performance based cognitive testing to measure executive functioning constructs. While BRIEF scores were noted to be, on average, somewhat lower than might be expected from the scale construction samples, subsequent studies have similarly noted that normative samples have mean calculated *t*-scores similar to the NCANDA sample (Roth et al., 2015). BRIEF scores were not related to age, sex, or race, and showed a significant relationship with SES. Prior studies have shown relationships between SES and executive functioning, with attributions of this correlation interpreted as related to parent education and other home and family environment characteristics (Hackman et al., 2015).

As expected, higher BRIEF scores were systematically correlated with NCANDA Risk Density. Previous studies have indicated that characteristics related to executive functioning are associated with SUD risk indicators, including externalizing characteristics (Familiar et al., 2015; Long et al., 2015), internalizing characteristics (Clark et al., 1997), family SUD history (Tapert and Brown, 2000), and the early adolescent onset of alcohol use (Tarter et al., 2003). However, the multivariate models indicated that the inclusion of BRIEF was accompanied by only a modest reduction in the relationship between NCANDA Risk Density and substance use, suggesting that the mediation hypothesis was not supported. These analyses of cross-sectionally collected data were not ideal for determining directional influences, and subsequent NCANDA analyses will be able to further examine the extent to which the relationship between risk factors and later substance use may be mediated by executive dysfunction.

Poor sleep quality was significantly and consistently correlated with all executive dysfunction dimensions. While prior research has suggested that sleep problems are associated with executive dysfunction in daily life, the robust correlations observed here indicates a more extensive relationship than has been previously reported. Since prior research utilizing BRIEF in relationship to sleep has involved smaller samples, the present finding may benefit from greater statistical power. The multivariate models indicated that the inclusion of BRIEF was accompanied by a reduction in the relationship between Sleep Quality and substance use, indicating that the mediation hypothesis was supported. Since the present findings are based on cross-sectional data, however, analyses informed by changes in these relationships over time will be undertaken in the course of the NCANDA study. In addition, polysomnography being examined in an NCANDA subset will complement subjective sleep assessments (Baker et al., 2016). Sleep difficulties have been shown to predict problematic alcohol use (Hasler and Clark, 2013; Hasler et al., 2014), and subsequent NCANDA analyses will be able to examine the extent to which executive dysfunction mediates this relationship over the course of adolescent development.

The self-report of executive dysfunction in daily life assessed by BRIEF were not significantly correlated with performance based cognitive test results. While one might expect there to be some correspondence between these indicators, our results are consistent with several prior studies. A recent review of 20 studies (Toplak et al., 2013) on the relationship between self-report and performance-based executive functioning assessments found that, of 286 relevant correlations, only 68 (24%) were statistically significant, and the median correlation was only 0.19. The authors concluded that these approaches generally assess different underlying constructs. The results here were consistent with these observations. Broadly conceived, performance-based cognitive tests and BRIEF scores assess executive functioning dimensions. However, our results, consistent with prior studies (Toplak et al., 2013), indicate that executive dysfunction in daily life, measured by BRIEF, and performance based cognitive testing measure distinct constructs.

BRIEF Global Composite scores were not significantly correlated with cortical structure or DTI indicators. These findings were consistent with some prior observations (e.g., Faridi et al., 2015). The current study had a substantially larger and more representative sample than some other pertinent prior studies with positive findings (Mahone et al., 2009; Clark et al., 2012). Alternative statistical approaches, such as latent variable modeling, may provide additional insights. For example, among 286 healthy children and adolescents, a latent variable modeling approach revealed a systematic pattern of relationships between cortical networks and BRIEF scores (Ziegler et al., 2013). The examination of network based brain activation patterns, using techniques such as resting state MRI, may show relationships between functional brain development and executive functioning difficulties (Fair et al., 2009; Casey et al., 2016). The neurobiological foundations of executive dysfunction may be more reflected in functional MRI indicators of activation during tasks that require cognitive and behavioral skills in this arena. For example, among 59 young adolescents (ages 12–15), BRIEF cognitive flexibility was associated with orbitofrontal cortex activation during a behavioral inhibition task (Zhai et al., 2015).

Consistent with prior studies (Clark et al., 2012; Hadjiefthyvoulou et al., 2012), BRIEF scores were associated with indicators of risky substance use, including a frequency of alcohol use indicating risk for AUD, age defined binge drinking, and marijuana use. Difficulties with executive functioning and risky substance use may reflect both preexisting psychological dysregulation and substance use resulting in disruption of executive functioning (Clark et al., 2012). While the cross-sectional structure of these NCANDA data from the initial assessment do not support interpretations on the direction of the observed associations, multivariate models were presented that may suggest directions for future studies. The age range in this study was broad, a feature which limits the size of subsamples representing each adolescent stage. For this examination of the initial assessment data, the sample was insufficient to subgroup subjects by age. When data collection for NCANDA has been completed, the acquisition of additional follow-up data in this accelerated longitudinal design and the application of statistical modeling techniques developed for such studies will support the examination of changes in these relationships over the course of adolescence. In addition, analyses with the longitudinal data will clarify the extent to which alternative hypotheses are reflected in observed relationships. While this sample size is larger than many other similar studies, the recruitment strategy and sample characteristics were designed to optimally provide data for later analyses taking advantage of the accelerated longitudinal design, and were not ideal for some of the analyses presented here.

In summary, these analyses found significant and systematic relationships between executive dysfunction in daily life and SUD risk indicators, including a composite risk indicator (i.e., NCANDA Risk Density), poor sleep quality, and risky alcohol and marijuana use. In addition, the study contributed to the available literature on the relationship between BRIEF scores and performance based cognitive testing, indicating that these methods assess complimentary but distinct constructs. Everyday executive functioning was not systematically related to cortical structure or white matter integrity by DTI. With the on-going collection and analysis of additional follow-up assessments with the measures described here, and the examination of functional brain characteristics and other variables, NCANDA will provide insights on the relationships among difficulties with everyday executive functioning, performance-based cognitive testing of skills reflecting executive functions, structural and functional brain development, and alcohol and other substance use effects on these neurodevelopmental trajectories.

These results suggest that executive dysfunction in daily life is not accounted for by deficits in the cognitive skills measured in the NCANDA protocol. This observation has clinical implications, in that the results here imply that such testing may not provide the expected insights into the origins of executive dysfunction. Also, executive dysfunction was not accounted for by cortical structural or DTI characteristics. While some support has been presented that cognitive tests relate to some related mental disorders (e.g., ADHD: Willcutt et al., 2005), these results are consistent with other negative studies in suggesting that cognitive testing and MRI studies may not provide clinical insights explaining executive dysfunction with typical adolescents.

Consistent with several prior studies (Hasler and Clark, 2013; Hasler et al., 2014, 2016, 2017), poor sleep quality was related to both executive dysfunction and risky substance use. These findings suggest interventions to improve sleep may benefit executive functioning as well as risky substance use. Individually applicable clinical interventions to improve sleep quality have demonstrated effectiveness in adolescents (Gradisar et al., 2014). On a more general level, early school times may be detrimental to sleep quality, later sleep times warrant consideration (Hasler et al., 2014; Minges and Redeker, 2016).

Interventions to improve executive functioning, in addition to having inherent value, may also be be a viable preventive intervention target. Interventions that improve inhibitory control in childhood, for example, hold promise for preventing SUD in adolescence (Riggs and Greenberg, 2009; Diamond and Lee, 2011).

## Ethics statement

This study was carried out in accordance with the recommendations of Human Subjects Institutional Review Boards as noted below with written informed consent from all subjects. Institutional review boards approved the study for each site. University of California, San Diego (UCSD) Human Subjects Institutional Review Boards approved the study at each site: University of California, San Diego; Oregon Health & Science University; SRI International; Duke University; University of Pittsburgh. The protocol was approved by these committees. All subjects gave written informed consent in accordance with the Declaration of Helsinki. For participants under 18 years old, a parent provided written informed consent and the under 18 participant provided assent. Participants 18 years old or older provided written informed consent.

## Author contributions

All authors (DC, TC, CM, BH, DF, BL, SB, ST, TB, KC, AP, ES, KP, IC, FB, MD, KN, and BN) were involved in study conception, data collection, and manuscript editing.

### Conflict of interest statement

The authors declare that the research was conducted in the absence of any commercial or financial relationships that could be construed as a potential conflict of interest.
